# Impact of Water Activity on the Inactivation and Gene Expression of *Listeria* *monocytogenes* during Refrigerated Storage of Pressurized Dry-Cured Ham

**DOI:** 10.3390/foods9081092

**Published:** 2020-08-10

**Authors:** Aida Pérez-Baltar, Alberto Alía, Alicia Rodríguez, Juan José Córdoba, Margarita Medina, Raquel Montiel

**Affiliations:** 1 Departamento de Tecnología de Alimentos, INIA, Carretera de La Coruña Km 7, 28040 Madrid, Spain; perez.aida@inia.es (A.P.-B.); mmedina@inia.es (M.M.); 2 Instituto Universitario de Investigación de Carne y Productos Cárnicos, Facultad de Veterinaria, Universidad de Extremadura, Avda. de la Universidad, s/n., 10003 Cáceres, Spain; albertoam@unex.es (A.A.); aliciarj@unex.es (A.R.); jcordoba@unex.es (J.J.C.)

**Keywords:** *L. monocytogenes*, high pressure processing, cured meat, qPCR, virulence and stress-related genes

## Abstract

*Listeria monocytogenes* population and the expression patterns of three virulence (*plcA*, *hly*, and *iap*) and one stress-related (*sigB*) genes in dry-cured ham with different water activity (a_w_) values (0.92, 0.88, and 0.84) and treated with high pressure processing (HPP, 450 MPa/10 min and 600 MPa/5 min) were monitored throughout 30 days (d) at 4 °C. The antimicrobial effect of HPP at 600 MPa against *L. monocytogenes* S4-2 (serotype 1/2b) and S12-1 (serotype 1/2c) was greater in dry-cured ham with a_w_ values of 0.92, with reductions of 2.5 and 2.8 log units, respectively. The efficacy of HPP treatments decreased at lower a_w_ values. Regarding gene expression, *L. monocytogenes* strains responded differently to HPP. For strain S4-2, the four target genes were generally overexpressed in dry-cured ham immediately after HPP treatments at the three a_w_ values investigated, although the extent of this induction was lower in the samples pressurized at 600 MPa and with a_w_ values of 0.84. For strain S12-1, the expression of all target genes was repressed at the three a_w_ values investigated. The antimicrobial efficacy of HPP against *L. monocytogenes* could be compromised by low a_w_ values in food products. However, no growth of HPP-survival cells was observed during refrigerated storage in low-a_w_ dry-cured ham, and the overexpression of virulence and stress-related genes decreased.

## 1. Introduction

*Listeria monocytogenes* is a foodborne pathogen causing listeriosis, mainly in neonates, the elderly, and pregnant or immunocompromised individuals. Although listeriosis has low morbidity, it is the most severe human disease in terms of hospitalization (more than 90% of cases) and has the highest mortality rate in the European Union, which was 15.6% in 2018 [1]. Certain strains of *L. monocytogenes* can form biofilms and withstand cleaning and disinfection processes allowing the pathogen to persist in the food processing installations for long periods. Moreover, this pathogen is able to overcome environmental stresses encountered in foods, such as refrigeration temperature (<5 °C), low pH (>4), or high content of NaCl (up to 12%). However, it is inhibited at water activity (a_w_) values below 0.92 [2].

Dry-cured ham can result contaminated with *L. monocytogenes* during post-processing steps (e.g., deboning or slicing). In the European Union, a maximum of 100 CFU/g of *L. monocytogenes* is allowed during the shelf-life for ready-to-eat (RTE) foods different to those intended for infants and medical purposes and that do not support the growth of the pathogen [3]. A “zero tolerance” criterion (absence in 25 g) is required in the USA [4], which is an impediment for the international commerce of cured meat products. The establishment of strategies to control *L. monocytogenes* (e.g., biopreservation and/or high pressure processing, HPP) would boost the international market of dry-cured ham.

HPP is a non-thermal technology effective to control foodborne pathogens and spoilage microorganisms, and its industrial implementation is continually growing. HPP can inactivate microorganisms subjecting the products to elevated pressures [5], controlling pathogens such as *L. monocytogenes* in dry-cured ham with minor changes in texture, color, or nutritional value [6]. HPP efficacy depends on various processing parameters such as the pressure level, temperature, and time of processing, as well as the type of microorganism and its growth phase. Pathogen lethality during HPP also depends very highly on the composition and the physico-chemical properties of the food matrix such as pH and a_w_ [7,8,9]. Low a_w_ values could reduce the antimicrobial efficacy of HPP against *L. monocytogenes* in sliced dry-cured ham and could result in changes in virulence and stress-related gene expression profiles of surviving *L. monocytogenes* cells [10,11].

Stress-related gene *sigB* contributes to overcome different conditions encountered in foods such as acidic, osmotic, or oxidative stresses [12] and to the adaptation of the pathogen to HPP [13]. Moreover, it contributes to the transcription of the *prfA* virulence gene cluster, the major virulence genetic locus identified in *L. monocytogenes* [14], which exerts a positive control on some virulence factors, including *plcA* and *hly* genes. Studies considering the effect of dry-cured ham a_w_ on the inactivation of *L. monocytogenes* by HPP are scarce and the consequences of the treatments for the physiology of the pathogen do not usually be considered. Current knowledge regarding the antilisterial effect of HPP in dry-cured ham should be integrated with information concerning molecular or cellular response of the pathogen to such treatments. Changes in gene expression may be of great help to understand changes in microbial physiology [15].

The aim of this work was to investigate the effect of dry-cured ham water activity on the inactivation and the relative expression of genes involved in virulence and stress response of two strains of *L. monocytogenes* during refrigerated storage after HPP treatment.

## 2. Materials and Methods

### 2.1. Microorganisms

Two *L. monocytogenes* strains (S4-2—serotype 1/2b and S12-1—serotype 1/2c) from the environment of a pork processing industry [16] were used as target organisms. Stock cultures in brain heart infusion (BHI, Biolife S.r.l., Milano, Italy) at −80 °C were subcultured twice onto Tryptic Soy Yeast Extract Broth (TSYEB, Biolife) at 37 °C for 18 h before use in experiments.

### 2.2. Adjustment of a_w_ Values in Sliced-Dry Cured Ham

One deboned dry-cured ham was obtained from a local supplier in Spain and aseptically sliced in a laminar flow cabinet (Bio II Advance, Telstar, Terrassa, Spain). The slices were then separately placed in pre-sterilized receptacles with saturated chloride solution in the bottom, where the relative humidity was maintained constant following the procedure previously reported by Andrade et al. [17]. In these receptacles, the slices of ham were kept during 10 h and a_w_ values were determined every hour by means of an Aqualab Series 3 instrument (Decagon Devices Inc., Pullman, WA, USA) at room temperature. Slices of three different a_w_ values were achieved in the receptacles: 0.92, 0.88, and 0.84, simulating hams of different aging times (drying, cellar, and finished product, respectively). Two independent trials with triplicate samples were performed.

### 2.3. Dry-Cured Ham Inoculation and HPP Treatments

After a_w_ adjustment, slices of 10 g were inoculated by spreading 100 μL of a cell suspension of *L. monocytogenes* S4-2 or S12-1 in order to achieve a final population of approximately 10^6^ CFU/g. Cell suspensions were prepared from overnight cultures in BHI broth. Ten gram non-inoculated slices were used to determine total viable counts (TVC) and pH. Inoculated and non-inoculated dry-cured ham slices were individually vacuum-packaged in high-barrier multilayer bags (BB325, Cryovac Sealed Air Corporation, Milan, Italy) and kept at 4 °C for 20 h. Samples were treated at 450 MPa for 10 min or 600 MPa for 5 min in a prototype Wave 6000/135 (NC Hyperbaric, Burgos, Spain). The compression rate was 218 MPa/min and the pressure release time was 6 s. The temperature of water used as pressure-transmitting medium was 19 °C. Sliced dry-cured ham, inoculated with either of the two *L. monocytogenes* strains, but not pressurized, was used as control. After treatments, all samples were kept at 4 °C during 30 days (d). Three independent experiments with duplicate samples were conducted.

### 2.4. Microbiological Analysis

Inoculated and non-inoculated samples were analyzed at 1, 15, and 30 d after HPP. Ten gram samples were 10-fold diluted with sterile 0.1% (*wt/vol*) peptone water solution and homogenized for 120 s in a stomacher (IUL Instruments, Barcelona, Spain). Enumeration of *L. monocytogenes* in inoculated samples was performed on duplicate plates of CHROMagar *Listeria* (CH-L, Scharlab S.l., Barcelona, Spain) incubated at 37 °C for 48 h. TVC in non-inoculated samples were enumerated in duplicate on Tryptic Soy Agar (TSA, Panreac Química SLU, Barcelona, Spain) incubated at 30 °C for 72 h.

### 2.5. Physicochemical Analysis

Non-inoculated samples were used to determine pH, sodium chloride (NaCl), and nitrites (NO^2-^) content of dry-cured ham. NaCl and NO^2−^ content were analyzed after the adjustment of a_w_ in non-pressurized dry-cured ham. pH determination was performed 1, 15, and 30 d after HPP in control and pressurized samples. Samples (5 g) were homogenized with 15 mL of distilled water for 120 s and pH was determined by means of a pH-meter GLP22 (Crison Instruments S.A., Barcelona, Spain). The reference method from ISO 2918:1975 [18] was used to evaluate nitrites content. The salt content was estimated as chloride using QUANTAB Chloride Titrator (Hach Company, Loveland, CO, USA) according to the Association of Official Analytical Chemists (AOAC) [19]. All analyses were performed in triplicate.

### 2.6. RNA Extraction and cDNA Synthesis

RNA extraction was performed at 0 h and 30 d after HPP. Samples were prepared as indicated in Section 2.4. One milliliter of the homogenates was used to extract total RNA using the MasterPure^™^ complete DNA and RNA purification kit (Epicentre, Madison, WI, USA) following the instructions of the manufacturer. A DNase treatment (Thermo Fisher Scientific, Walthman, MA, USA) was done to digest residual genomic DNA. Then, RNA concentration and purity were determined using a Nanodrop^™^ (Thermo Fisher Scientific) and normalized to 100 ng/µL. Reverse transcription reaction for cDNA synthesis was conducted in a Mastercycler^®^ EP (Eppendorf Scientific, Hamburg, Germany) using the PrimeScript^™^ Reagent (Perfect Real Time) kit (TaKaRa Bio Inc., Dalian, China). Approximately, 5 µg of total RNA were retrotranscripted at 37 °C for 15 min. The reaction was stopped by inactivation of the enzyme at 85 °C for 5 s. Finally, cDNA was kept at −20 °C until use.

### 2.7. Quantitative PCR

Quantitative PCR (qPCR) based on TaqMan^®^ methodology was used to amplify the virulence-associated genes *plcA, hly,* and *iap*, and stress-related gene *sigB* of *L. monocytogenes* (Table 1) as previously described Alía et al. [10]. *IGS* was selected as a reference gene and internal control. Two biological replicates were analyzed for each gene of interest and each sample was amplified in triplicate in MicroAmp Fast Optical 96-Well Reaction Plate (0.1 mL) (Thermo Fisher Scientific, Walthman, MA, USA). qPCR was carried out using the qPCR Vii™ 7 System (Thermo Fisher Scientific, Walthman, MA, USA). Reactions (final volume of 12.5 µL) contained: 2.5 µL of cDNA templates, 7 µL of Premix ExTaq^TM^ (TaKaRa Bio Inc., Dalian, China), 0.125 µL of 50x ROX^TM^ Reference Dye (TaKaRa Bio Inc., Dalian, China), 300 nM (*IGS, plcA*, *iap*, and *sigB*), or 450 nM (*hly*) of each primer, and 100 nM (*IGS, hly*), 200 nM (*plcA*), or 300 nM (*iap* and *sigB*) of the probe. The amplification program consisted of one cycle at 95 °C for 10 min, followed by 40 cycles of 15 s at 95 °C, 1 min at 55 °C (*IGS* and *plcA*), or 60 °C (*hly, iap,* and *sigB*). 

### 2.8. Data and Statistical Analysis

Threshold cycle (*C_T_*) values from qPCR were used for relative quantification. Mean *C_T_* values for each analysis condition were obtained to calculate the relative gene transcription levels (fold changes) by the 2^-ΔΔ*CT*^ method, where ΔΔ*C_T_* is (*C_T_*_target_ − *C_T_*_reference gene_)_test condition_ − (*C_T_*_target_ − *C_T_*_reference gene_)_control condition_ [21]. Stress or virulence genes were considered targets, while *IGS* was considered a reference gene whose transcription is considered stable even under experimental treatments. Control condition corresponded to non-pressurized dry-cured ham, while test condition corresponded to pressurized dry-cured ham, at the different a_w_ values and the respective time points.

SPSS Statistics 22.0 software (IBM Corp., Armonk, NY, USA) was used to carry out the statistical treatment of log_2_ values of relative gene expression as well as to evaluate the significant differences between *L. monocytogenes* counts, TVC, and pH values. The Tukey test was applied to detect significant differences between means at α = 0.05 [22].

## 3. Results

### 3.1. Adjustment of a_w_ in Sliced Dry-Cured Ham

**Figure 1:**
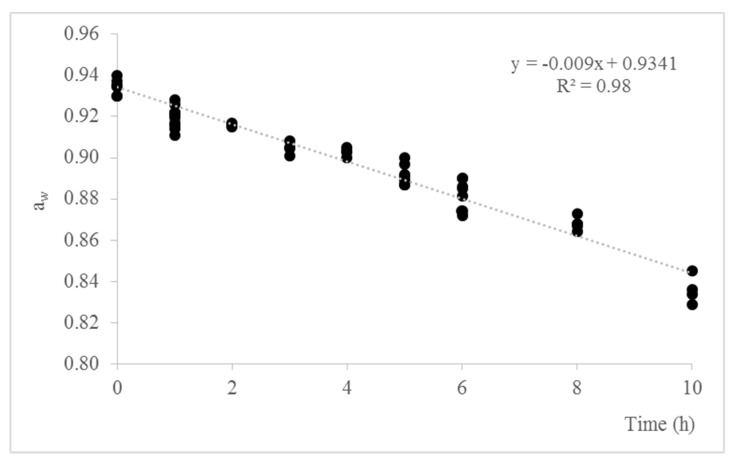
Adjustment of a_w_ values in sliced dry-cured ham after exposition to saturated chloride solution in sterilized receptacles during 10 h.

Figure 1 shows the decrease of a_w_ values in sliced dry-cured ham over time. The initial a_w_ was 0.935 ± 0.004. Slices of dry-cured ham reached a_w_ values of 0.92, 0.88, and 0.84 after 1, 6, and 10 h exposed to saturated chloride solution, respectively. NaCl and NO^2-^ content of dry-cured ham after adjustment of a_w_ are indicated in Table 2. No statistical differences in NaCl and NO^2-^ content were detected as consequence of a_w_ modification. NaCl and NO^2-^ values ranged from 3.6 to 5.3% and from 3.8 to 4.3 mg/kg, respectively.

### 3.2. Effect on L. monocytogenes Population

**Figure 2:**
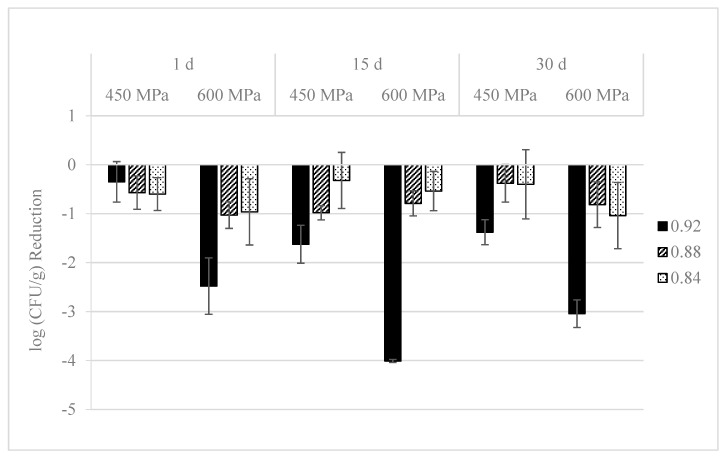
*L. monocytogenes* S4-2 inactivation (log CFU/g reduction) in sliced dry-cured ham with different a_w_ values (0.92, 0.88, and 0.84) treated with HPP (450 MPa/10 min or 600 MPa/5 min) and stored during 30 days at 4 °C. All values represent the average of three independent experiments with duplicate samples. Error bars designate standard deviation.

**Figure 3:**
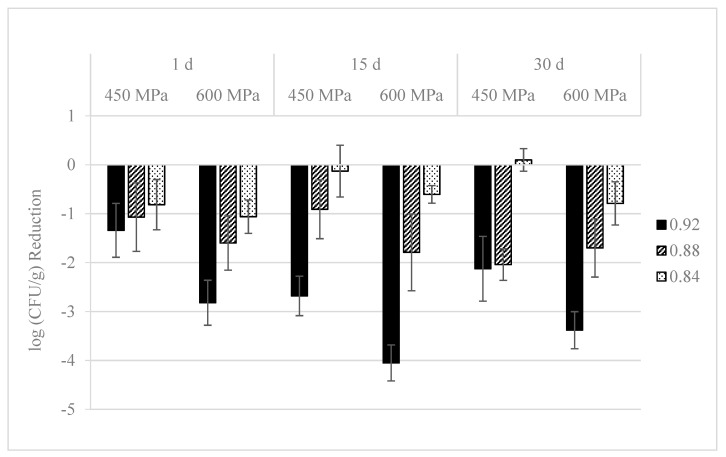
*L. monocytogenes* S12-1 inactivation (log CFU/g reduction) in sliced dry-cured ham with different a_w_ values (0.92, 0.88, and 0.84) treated with HPP (450 MPa/10 min or 600 MPa/5 min) and stored during 30 days at 4 °C. All values represent the average of three independent experiments with duplicate samples. Error bars designate standard deviation.

Reductions on *L. monocytogenes* S4-2 and S12-1 population in pressurized dry-cured ham with different a_w_ values during refrigerated storage at 4 °C are shown in Figure 2 and Figure 3, respectively. One day after HPP, strain S4-2 levels were 6.6, 6.1, and 6.1 log CFU/g in non-pressurized dry-cured ham with 0.92, 0.88, and 0.84 a_w_ values, respectively. Reductions of *L. monocytogenes* by pressurization at 450 MPa were lower than 1.0 log in dry-cured ham with different a_w_ values. The inactivation of *L. monocytogenes* S4-2 was significantly (*p* ≤ 0.001) higher at 600 MPa/5 min, with reductions of 2.5 log units in samples with the highest (0.92) a_w_ value. The antimicrobial activity of such pressure treatment decreased (*p* ≤ 0.05) when it was applied in dry-cured ham with lower (0.88 and 0.84) a_w_ values, with levels 1.0 log units lower than in non-pressurized samples, respectively.

Regarding *L. monocytogenes* S12-1, initial counts were 6.5, 6.1, and 5.8 log CFU/g in control dry-cured ham with a_w_ values of 0.92, 0.88, and 0.84, respectively. Reductions lower than 1.5 log units were achieved when 450 MPa/10 min were applied in dry-cured ham with different a_w_. HPP at 600 MPa/5 min resulted in higher reductions (*p* ≤ 0.001) in the samples with the highest (0.92) a_w_ value, with counts 2.8 log units lower than those observed in non-pressurized dry-cured ham. The antilisterial effect of such treatment diminished (*p* ≤ 0.05) when it was applied in samples with lower (0.88 and 0.84) a_w_, with levels 1.8 and 1.1 log CFU/g lower than those in non-pressurized ham, respectively.

During refrigeration storage, *L. monocytogenes* S4-2 and S12-1 counts kept stable in control (non-pressurized) 0.92 a_w_ samples, with counts after 30 d approximately 0.2 log units lower than those observed at day 1. Pathogen counts decreased slightly during storage in dry-cured ham with 0.88 and 0.84 a_w_ values, with differences between strains. The reduction was 0.5 log units after 30 d and a_w_ values of 0.88 and 0.84 for strain S4-2, and 0.3 and 1.0 log units at 0.88 and 0.84, respectively, for strain S12-1.

Strain S4-2 levels significantly (*p* ≤ 0.05) diminished throughout storage in samples pressurized at 450 and 600 MPa and a_w_ of 0.92, with counts 1.4 and 3.0 log units lower, respectively, than those observed in control ham at day 30, whereas kept invariable in samples with lower (0.88 and 0.84) a_w_. Concerning strain S12-1, levels also diminished (*p* ≤ 0.05) throughout the refrigerated storage of cured ham with a_w_ of 0.92 and pressurized at 450 and 600 MPa. Counts were 2.1 and 3.4 log CFU/g lower than those observed in non-pressurized control ham at day 30, respectively. In samples with a_w_ of 0.88, these reductions were 2.0 and 1.7 log units, respectively, whereas in dry-cured ham with a_w_ of 0.84, pathogen levels did not change during the storage.

### 3.3. Effect on TVC and pH

**Figure 4:**
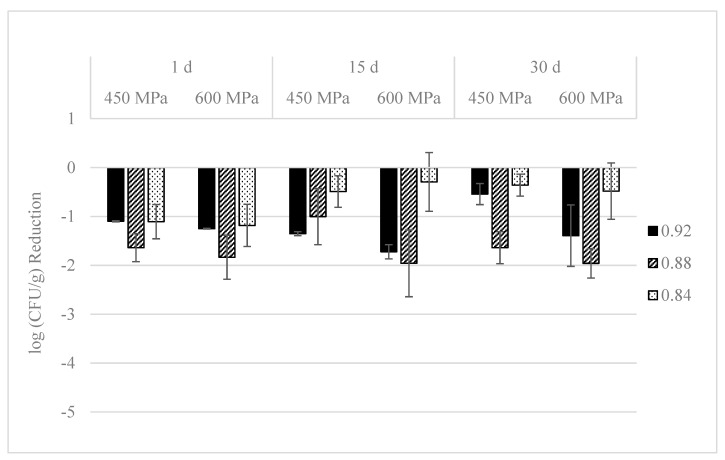
Total viable counts (TVC, log CFU/g reduction) in sliced dry-cured ham with different a_w_ values (0.92, 0.88, and 0.84) treated with HPP (450 MPa/10 min or 600 MPa/5 min) and stored during 30 days at 4 °C. All values represent the average of three independent trials with duplicate samples. Error bars designate standard deviation.

Reductions on TVC in control and pressurized dry-cured ham with different a_w_ values during storage at 4 °C are represented in Figure 4. At day 1, TVC in control samples were 5.4, 5.7, and 6.1 log CFU/g in dry-cured ham with 0.92, 0.88, and 0.84 a_w_ values, respectively. HPP significantly (*p* ≤ 0.05) diminished microbial levels in dry-cured ham with different a_w_ values. Reductions of TVC by 450 MPa/10 min were 1.1, 1.7, and 1.1 log units in samples with a_w_ values of 0.92, 0.88, and 0.84, respectively. Such reductions by 600 MPa/5 min were 1.3, 1.9, and 1.1 log CFU/g, respectively. During refrigerated storage, TVC slightly increased (*p* ≤ 0.05) in non-pressurized dry-cured ham with a_w_ values of 0.92, but this growth was avoided by HPP. TVC population kept unchanged in pressurized and non-pressurized samples with 0.88 and 0.84 a_w_ values throughout 30 d of storage.

Values of pH in control and pressurized dry-cured ham with different a_w_ values during storage at 4 °C are indicated in Table 3. One day after HPP, pH values ranged from 5.82 to 6.01 in control and pressurized dry-cured ham with 0.92–0.84 a_w_ values. There were no significant differences in pH values when pressure was applied in dry-cured ham with different a_w_ values. During refrigeration, values of pH kept constant in control and treated dry-cured ham with higher a_w_ values, whereas a slight decrease (*p* ≤ 0.05) was registered in samples with a_w_ of 0.88 and 0.84, with values not lower than 5.78 after 30 d at 4 °C.

### 3.4. Effect on L. monocytogenes Gene Expression

**Figure 5:**
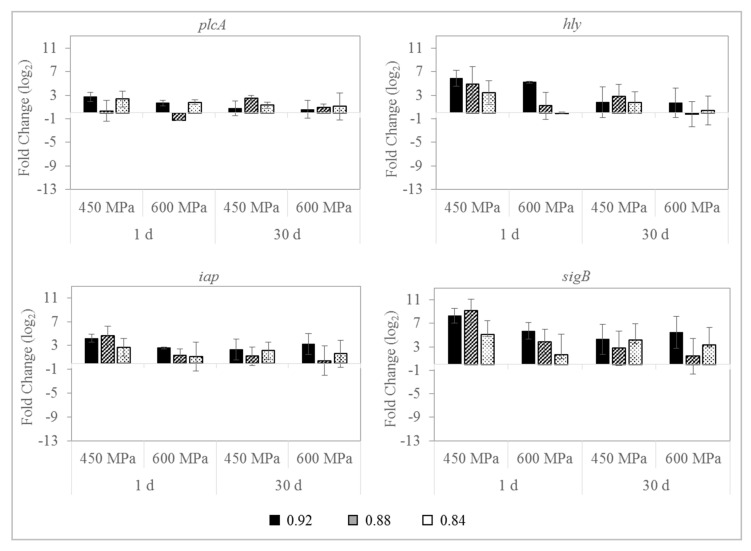
Relative changes in the transcription level for virulence (*plcA, hly,* and *iap*) and stress-related (*sigB*) genes of *L. monocytogenes* strain S4-2 (serotype 1/2b) in sliced dry-cured ham with different a_w_ values (0.92, 0.88, and 0.84), treated with HPP (450 MPa/10 min or 600 MPa/5 min) and stored during 30 days at 4 °C. Error bars indicate standard deviation of three biological replicates with duplicated samples.

**Figure 6:**
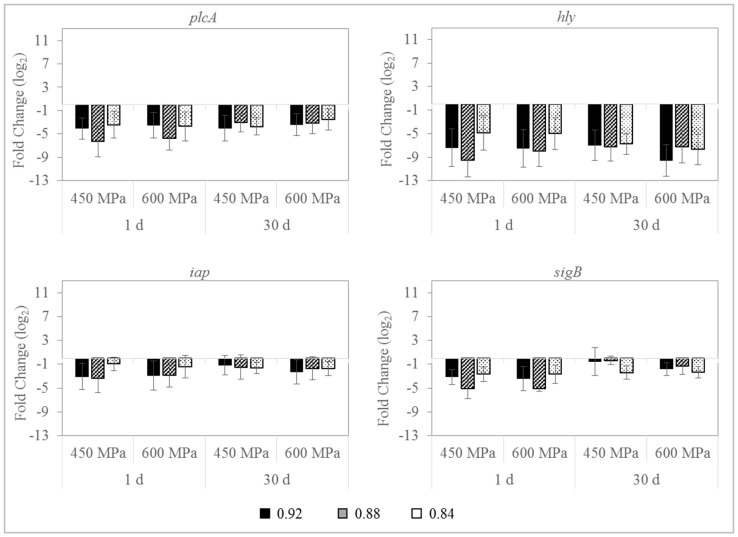
Relative changes in the transcription level for virulence (*plcA, hly,* and *iap*) and stress-related (*sigB*) genes of *L. monocytogenes* strain S12-1 (serotype 1/2c) in sliced dry-cured ham with different a_w_ values (0.92, 0.88, and 0.84) treated with HPP (450 MPa/10 min or 600 MPa/5 min) and stored during 30 days at 4 °C. Error bars indicate standard deviation of three biological replicates with duplicated samples.

The relative gene transcription profiles of four virulence and stress-related genes (*plcA, hly, iap,* and *sigB*) of *L. monocytogenes* S4-2 and S12-1 in sliced dry-cured ham with different a_w_ (0.92, 0.88, and 0.84) and treated with HPP (450 MPa/10 min or 600 MPa/5 min) during 30 d at 4 °C are shown in Figure 5 and Figure 6, respectively. Relative gene expression profiles of the two *L. monocytogenes* strains belonging to different serotype were significantly different. For strain S4-2 (serotype 1/2b), an overall upregulation was registered in dry-cured ham at the three a_w_ values investigated, immediately after HPP, which was more noticeable for *hly* and *sigB* genes. Generally, such overexpression was higher in samples with higher a_w_ values and pressurized at 450 MPa/10 min, being statistically significant (*p* ≤ 0.05) for *sigB* and *iap* genes. Contrary, for strain S12-1 (serotype 1/2c), all target genes were significantly repressed (*p* ≤ 0.01) immediately after pressurization, being more pronounced for *hly* and *plcA* genes. This downregulation was greater in pressurized dry-cured ham with 0.92 and 0.88 a_w_ values, with fold changes higher than nine for *hly* gene.

The expression of the target genes fluctuated during refrigeration storage. For strain S4-2, the overexpression initially recorded for all genes was reduced during the 30 d of the refrigerated storage, which was generally lower in dry-cured ham with 0.88 and 0.84 a_w_ values and subjected to 600 MPa. For S12-1 strain, the initial downregulation was also diminished throughout the storage at 4 °C, mainly for *sigB* gene.

## 4. Discussion

The water activity of dry-cured ham was modified to attain values of 0.92, 0.88, and 0.84, simulating hams of different aging times (drying, cellar, and finished products, respectively). Two strains of *L. monocytogenes* were subjected to HPP in dry-cured ham with different a_w_ values, strain S4-2 (serotype 1/2b) which was considered a persistent strain found in the environment and raw and final cured meat products and strain S12-1 (serotype 1/2c), which was non-persistent and isolated from dry-cured products [16]. Reductions on *L. monocytogenes* S4-2 population attained by 600 MPa/5 min were 2.5 log units in dry-cured ham with higher a_w_ values (0.92). Strain S12.1 resulted significantly (*p* ≤ 0.05) more sensitive to HPP, with reductions higher than 2.8 log units. The efficacy of the treatments against *L. monocytogenes* diminished at lower a_w_ values mainly at 600 MPa/5 min. Thus, reductions in S4-2 and S12-1 population were equal or less than 1.0 and 1.6 log units, respectively, when 600 MPa/5 min treatments were applied to dry-cured ham with a_w_ values of 0.88 and 0.84. The efficacy of HPP to eliminate *L. monocytogenes* in dry-cured ham has been reported to be affected by physico-chemical properties of the food matrix such as a_w_ that could be very variable in this product. Thus, according to Bover-Cid et al. [23], dry-cured ham a_w_ can vary from 0.85 and 0.94. In vacuum-packed sliced dry-cured ham samples, up to 60% presented values of 0.92 or higher [24]. Low a_w_ values in dry-cured ham could protect microorganisms against HPP and diminish the bacterial inactivation caused by these treatments [25]. *L. monocytogenes* population was reduced 3.85 log units by 600 MPa/5 min in dry-cured hams with a_w_ values of 0.92, whereas reductions of 1.85 log units were obtained in hams with a longer drying and ripening period and a_w_ values of 0.88 [24]. This decrease in the antilisterial effect of HPP was also registered by Bover-Cid et al. [23], who observed reductions of 6.8 and 2.2 log units by 600 MPa/5 min in dry-cured ham with a_w_ values of 0.96 and 0.86, respectively, and by Morales et al. [26], who reported higher inactivation of *L. monocytogenes* in Iberian dry-cured ham (a_w_ = 0.90) pressurized at 450 MPa/10 min than in Serrano ham (a_w_ = 0.88).

The predominant microorganisms in low a_w_ and high salt content foods as dry-cured ham are Gram-positive, catalase-positive cocci, as well as molds and yeasts [27]. Only microbial groups adapted to the low a_w_ values will survive during the ripening of dry-cured ham. In the present work, reductions on TVC ranged from 1.1 to 1.9 log units in dry-cured ham with a_w_ values of 0.92, 0.88, and 0.84 and subjected to 600 MPa/5 min, being greater at 0.92 a_w_ values. Moreover, bacterial counts increased in non-pressurized dry-cured ham with a_w_ values of 0.92 throughout the storage, whereas kept unchanged or even decreased in samples with a_w_ values of 0.88 and 0.84, respectively. Total aerobic counts were reduced by 1.2 log units in dry-cured ham with a_w_ values of 0.89 and treated at 600 MPa for 6 min [28]. Contrary, Martínez-Onandi et al. [29] concluded that a_w_ did not significantly change TVC in either untreated or dry-cured ham treated at 600 MPa/6 min.

Values of pH in dry-cured ham were barely affected as consequence of a_w_ modification or HPP, being the differences between samples lower than 0.2 pH units. Values of a_w_ neither affected nitrites nor NaCl content in dry-cured ham. Previous works have observed that HPP caused minor changes in the pH of dry-cured ham [30,31].

The influence of HPP on gene transcription patterns of *L. monocytogenes* in real food products has been scarcely studied. Moreover, the role of physicochemical properties of dry-cured ham such as a_w_ on the physiology of the pathogen has not been adequately approached. Therefore, one purpose of this work was to evaluate the impact of a_w_ on the relative expression of four genes involved in virulence and the stress response (*plcA*, *hly*, *iap*, and *sigB*) of *L. monocytogenes* inoculated in dry-cured ham and subjected to HPP. Moreover, relative gene transcription was compared for two strains of *L. monocytogenes* belonging to different serotypes. Our results suggest that changes in HPP-surviving bacteria gene transcription patterns were strain-dependent. In strain S4-2 (serotype 1/2b) the target genes *plcA*, *hly*, *iap*, and *sigB* were generally overexpressed by HPP in dry-cured ham at the three a_w_ values investigated, while in strain S12-1 (serotype 1/2c) the above genes were repressed. The differential behavior of *L. monocytogenes* strains was previously reported after the exposure of the pathogen to an enterocin extract or bacteriocin-producing *Enterococcus faecalis* in dry-cured ham [32], and when 3.5% of NaCl was used in a cheese-based medium to control *L. monocytogenes* [33].

*L. monocytogenes* potential to respond to adverse conditions or changes in the environment is mediated by various alternative sigma (σ) factors. The alternative sigma factor, SigB (σ^B^), allows *L. monocytogenes* to multiply and survive under stress conditions in non-host associated environments [34], including those encountered in foods such as acidic or osmotic conditions [13,35]. In this work, an upregulation of *sigB* gene expression was observed for strain S4-2 in pressurized dry-cured ham with different a_w_, being such induction smaller at lower a_w_ values. In this way, an increase of the *sigB* expression levels was reported in dry-cured fermented sausages, with decreasing values of pH and a_w_ during the manufacturing process [36]. Similarly, the activity of the SigB of *L. monocytogenes* was induced when the pathogen was exposed to 3% NaCl (≈ 0.96 − 0.97 a_w_) [37]. Contrary, a downregulation of *sigB* was recorded for strain S12-1, being the expression also less pronounced in samples with the lowest a_w_ values. HPP-surviving cells could be highly injured, which may lead to increase in the expression of cell damage repair mechanisms and to repress virulence genes [38], mainly in dry-cured ham with very low a_w_ values.

SigB contributes to overcome food processing such as HPP [13], is fundamental in stress regulation and mediates the transcription of PrfA, the major regulator of *Listeria* virulence. The repression of *sigB* and *prfA* genes was observed for *L. monocytogenes* S12-1 when 450 MPa/10 min or 600 MPa/5 min were applied in dry-cured ham with different a_w_ values. Similarly, Bowman et al. [38] reported the suppression of these genes for a *L. monocytogenes* strain belonging to serotype 1/2a when HPP at 400 or 600 MPa for 5 min was applied on TSYE broth. On the contrary, *sigB* gene expression was increased after HPP for strain S4-2, but such initial overexpression was attenuated throughout the storage at 4 °C.

PrfA mediates the transcription of several virulence genes, including *plcA* and *hly*. Thus, a downregulation of *sigB* gene could result in a repression of *prfA* and, consequently, in reducing levels of *plcA* and *hly*. This fact could explain the trend of expression of these genes, which was similar to that observed for *sigB* gene. Immediately after pressure treatments, *hly* and *plcA* genes were upregulated for S4-2 strain in dry-cured ham with 0.92, 0.88, and 0.84 a_w_ values and repressed for S12-1. Same tendency was registered for *iap* gene, which encoded the invasion-associated protein p60 and is involved in the invasion of mammalian cells [39].

*L. monocytogenes* S4-2 and S12-1 responded differently to HPP. Strain S12-1 was slightly more sensitive to pressurization treatments than strain S4-2. HPP-surviving cells of strain S12-1 could result in more injured and this might have led to increased expression of cell damage repair genes but not virulence or stress-related genes. In the same way, pressure treatments at 400 or 600 MPa for 5 min caused a downregulation of genes associated with growth and virulence, such as *hly* and *iap*, for a *L. monocytogenes* strain (serotype 1/2a) in TSYE broth [38]. Differences in response to HPP and gene expression between strains might be related with serotype and/or other characteristics as persistence in the industrial environment. Further studies would be necessary to elucidate the differences observed in the present work.

## 5. Conclusions

*L. monocytogenes* strains S4-2 (serotype 1/2b) and S12-1 (serotype 1/2c) were reduced greatly by HPP in sliced dry-cured ham with a_w_ values of 0.92, mainly at 600 MPa/5 min. The efficacy of the HPP treatments diminished at lower a_w_. Pressure treatments and a_w_ values of dry-cured ham changed the expression patterns of four virulence and stress-related genes (*plcA, hly, iap,* and *sigB*) but the behavior of two strains of the pathogen was different. *L. monocytogenes* S4-2 exhibited an upregulation of the target genes involved in virulence and stress-related, mainly in dry-cured ham with a_w_ values of 0.92, which was attenuated during refrigerated storage. Strain S12-1 showed a downregulation of all genes tested after HPP, mainly for *hly* and *plcA*. This study highlights that HPP effectiveness against *L. monocytogenes* could be reduced by low a_w_ values and that gene expression may be influenced by HPP and a_w_ values in dry-cured ham. However, no growth of HPP-survival cells was observed during refrigerated storage in low-a_w_ dry-cured ham, and the overexpression of virulence and stress-related genes diminished. The a_w_ values and other physicochemical properties such as NaCl or nitrites content in dry-cured ham is very variable and could hardly influence the stress adaptation mechanisms of the pathogen. Therefore, a better understanding of the role of such physicochemical properties of dry-cured ham on the physiology of *L. monocytogenes* should be further evaluated.

## Figures and Tables

**Table 1:** *L. monocytogenes* genes targeted by qPCR in this study.

Gene	Function and Scope of Use	Sequence (5′→3′)	Reference
*IGS*	Reference gene	F: GGCCTATAGCTCAGCTGGTTA	Rantsiou et al. 2012 [14]
		R: GCTGAGCTAAGGCCCCATAAA	
		P: FAM-ATAAGAAATACAAATAATCATACCCTTTTAC-TAMRA	
*plcA*	Escape from primary vacuoles	F: CTAGAAGCAGGAATACGGTACA	Rantsiou et al., 2012 [14]
		R: ATTGAGTAATCGTTTCTAAT	
		P: HEX-AATTTATTTAAATGCATCACTTTCAGGT-TAMRA	
*hly*	Lysis of vacuoles	F: CATGGCACCACCAGCATCT	Rodríguez-Lázaro et al., 2004 [20]
		R: ATCCGCGTGTTTCTTTTCGA	
		P: HEX-CGCCTGCAAGTCCTAAGACGCCA-TAMRA	
*iap*	Invasion associated secreted endopeptidase	F: AATCTTTAGCGCAACTTGGTTAA	Rodríguez-Lázaro et al., 2004 [20]
		R: CACCTTTGATGGACGTAATAATACTGTT	
		P: HEX-CAACACCAGCGCCACTAGGACG-TAMRA	
*sigB*	Transcription factor, regulation of virulence and stress-response genes	F: CCAAGAAAATGGCGATCAAGAC	Rantsiou et al., 2012 [14]
		R: CGTTGCATCATATCTTCTAATAGCT	
		P: HEX-TGTTCATTACAAAAACCTAGTAGAGTCCAT-TAMRA	

F, forward; R, reverse; P, probe; FAM/HEX, fluorochrome at 5′-end of the probe; and TAMRA, quencher of FAM/HEX at 3′-end of the probe.

**Table 2:** Physicochemical characteristics of sliced dry-cured ham with different a_w_ values.

a_w_	NaCl (% *wt/wt*)	Nitrites (mg/kg)
0.92	3.6 ± 0.3 a	3.9 ± 0.3 a
0.88	4.3 ± 1.3 a	3.8 ± 0.0 a
0.84	5.3 ± 0.0 a	4.3 ± 0.2 a

Values are means ± SD. Values in the same column with different lowercase indicate significant differences at *p* ≤ 0.05.

**Table 3:** Values of pH in sliced dry-cured ham with different a_w_ values (0.92, 0.88, and 0.84) and treated with HPP (450 MPa/10 min or 600 MPa/5 min) during 30 days at 4 °C.

a_w_	Treatment	Time (d)		
		1	15	30
0.92	NP	5.82 ± 0.04 aA	5.82 ± 0.08 aA	5.89 ± 0.07 aA
	450 MPa/10 min	5.88 ± 0.12 aA	5.91 ± 0.08 aA	5.87 ± 0.07 aA
	600 MPa/5 min	5.95 ± 0.08 aA	6.06 ± 0.07 bB	5.85 ± 0.02 aA
0.88	NP	5.88 ± 0.07 aB	6.07 ± 0.06 bC	5.78 ± 0.06 aA
	450 MPa/10 min	5.86 ± 0.13 aAB	5.98 ± 0.05 aB	5.80 ± 0.05 aA
	600 MPa/5 min	5.92 ± 0.02 aB	5.96 ± 0.04 aB	5.86 ± 0.06 aA
0.84	NP	5.93 ± 0.08 aA	6.12 ± 0.05 aB	5.86 ± 0.03 abA
	450 MPa/10 min	6.01 ± 0.12 aA	6.12 ± 0.02 aB	5.91 ± 0.03 bA
	600 MPa/5 min	5.97 ± 0.07 aB	6.14 ± 0.12 aC	5.83 ± 0.05 aA

Values are means ± SD. NP, non-pressurized. Means within the same column with different lowercase indicate significant differences at *p* ≤ 0.05 for a given a_w_. Values in the same row with different uppercase indicate significant differences at *p* ≤ 0.05.

## References

[1] European Food Safety Authority (EFSA) (2019). Scientific report on the European Union One Health 2018 Zoonoses Report. EFSA J..

[2] Lado B., Yousef A.E., Ryser E.T., Marth E.H. (2007). Characteristics of *Listeria monocytogenes* important to food processors. Listeria, Listeriosis and Food Safety.

[3] European Commission (EC) (2005). Commission regulation (EC) No 2073/2005 of 15 November 2005 on microbiological criteria for foodstuffs. Off. J. Eur..

[4] Food Safety and Inspection Service (USDA-FSIS) (2014). FSIS Compliance Guideline: Controlling Listeria monocytogenes in Post-Lethality Exposed Ready-to-Eat Meat and Poultry Products. https://www.fsis.usda.gov/wps/wcm/connect/d3373299-50e6-47d6-a577-e74a1e549fde/Controlling-Lm-RTE-Guideline.pdf?MOD=AJPERES.

[5] Zhu M., Du M., Cordray J., Ahn D.U. (2005). Control of *Listeria monocytogenes* contamination in ready-to-eat meat products. Compr. Rev. Food Sci. Food Saf..

[6] Hugas M., Garriga M., Monfort J.M. (2002). New mild technologies in meat processing: High pressure as a model technology. Meat Sci..

[7] Campus M. (2010). High Pressure Processing of Meat, Meat Products and Seafood. Food Eng. Rev..

[8] Bover-Cid S., Belletti N., Aymerich T., Garriga M. (2017). Modelling the impact of water activity and fat content of dry-cured ham on the reduction of *Salmonella enterica* by high pressure processing. Meat Sci..

[9] Rubio B., Possas A., Rincón F., García-Gímeno R.M., Martínez B. (2018). Model for *Listeria monocytogenes* inactivation by high hydrostatic pressure processing in Spanish chorizo sausage. Food Microbiol..

[10] Ferreira M., Almeida A., Delgadillo I., Saraiva J., Cunha A. (2016). Susceptibility of *Listeria monocytogenes* to high pressure processing: A review. Food Rev. Int..

[11] Alía A., Rodríguez A., Andrade M.J., Gómez F.M., Córdoba J.J. (2019). Combined effect of temperature, water activity and salt content on the growth and gene expression of *Listeria monocytogenes* in a dry-cured ham model system. Meat Sci..

[12] Dorey A., Marinho C., Piveteau P., O’Byrne C. (2019). Role and regulation of the stress activated sigma factor sigma B (σ^B^) in the saprophytic and host-associated life stages of *Listeria monocytogenes*. Adv. Appl. Microbiol..

[13] Wemekamp-Kamphuis H.H., Wouters J.A., De Leeuw P.P.L.A., Hain T., Chakraborty T., Abee T. (2004). Identification of sigma factor σ^B^-controlled genes and their impact on acid stress, high hydrostatic pressure, and freeze survival in *Listeria monocytogenes* EGD-e. Appl. Environ. Microbiol..

[14] Rantsiou K., Mataragas M., Alessandria V., Cocolin L. (2012). Expression of virulence genes of *Listeria monocytogenes* in food. J. Food Saf..

[15] Greppi A., Rantsiou K. (2016). Methodological advancements in foodborne pathogen determination: From presence to behavior. Curr. Opin. Food Sci..

[16] Ortiz S., López V., Villatoro D., López P., Dávila J.C., Martínez-Suárez J.V. (2010). A 3-year surveillance of the genetic diversity and persistence of *Listeria monocytogenes* in an Iberian pig slaughterhouse and processing plant. Foodborne Pathog. Dis..

[17] Andrade M.J., Thorsen L., Rodríguez A., Córdoba J.J., Jespersen L. (2014). Inhibition of ochratoxigenic moulds by *Debaryomyces hansenii* strains for biopreservation of dry-cured meat products. Int. J. Food Microbiol..

[18] International Organization for Standardization (1975). Meat and Meat Products. Determination of Nitrite Content, ISO 2918.

[19] Association of Official Analytical Chemists (2012). Official Methods of Analysis.

[20] Rodríguez-Lázaro D., Hernández M., Scortti M., Esteve T., Vázquez-Boland J.A., Pla M. (2004). Quantitative detection of *Listeria monocytogenes* and *Listeria innocua* by Real-Time PCR: Assessment of *hly*, *iap*, and *lin02483* targets and AmpliFluor technology. Appl. Environ. Microbiol..

[21] Livak K.J., Schmittgen T.D. (2001). Analysis of relative gene expression data using real-time quantitative PCR and the 2^-ΔΔCT^ method. Methods.

[22] Steel R.G.D., Torrie J.H., Dickey D. (1996). Principles and Procedures of Statistics: A Biometrical Approach.

[23] Bover-Cid S., Belletti N., Aymerich T., Garriga M. (2015). Modeling the protective effect of a_w_ and fat content on the high pressure resistance of *Listeria monocytogenes* in dry-cured ham. Food Res. Int..

[24] Hereu A., Bover-Cid S., Garriga M., Aymerich T. (2012). High hydrostatic pressure and biopreservation of dry-cured ham to meet the Food Safety Objectives for *Listeria monocytogenes*. Int. J. Food Microbiol..

[25] Patterson M.F. (2005). Microbiology of pressure-treated foods. J. Appl. Microbiol..

[26] Morales P., Calzada J., Nuñez M. (2006). Effect of high-pressure treatment on the survival of *Listeria monocytogenes* Scott A in sliced vacuum-packaged Iberian and Serrano cured hams. J. Food Prot..

[27] Rodríguez M., Núñez F., Córdoba J.J., Bermúdez M.E., Asensio M.A. (1998). Evaluation of proteolytic activity of microorganisms isolated from dry cured ham. J. Appl. Microbiol..

[28] Garriga M., Grèbol N., Aymerich M.T., Monfort J.M., Hugas M. (2004). Microbial inactivation after high-pressure processing at 600 MPa in commercial meat products over its shelf life. Innov. Food Sci. Emerg. Technol..

[29] Martínez-Onandi N., Castioni A., San Martín E., Rivas-Cañedo A., Nuñez M., Torriani S., Picon A. (2017). Microbiota of high-pressure-processed Serrano ham investigated by culture-dependent and culture-independent methods. Int. J. Food Microbiol..

[30] Pérez-Baltar A., Serrano A., Bravo D., Montiel R., Medina M. (2019). Combined effect of high pressure processing with enterocins or thymol on the inactivation of *Listeria monocytogenes* and the characteristics of sliced dry-cured ham. Food Bioprocess Technol..

[31] Pérez-Baltar A., Serrano A., Montiel R., Medina M. (2020). *Listeria monocytogenes* inactivation in deboned dry-cured hams by high pressure processing. Meat Sci..

[32] Montiel R., Quesille-Villalobos A., Alessandria V., Medina M., Cocolin L.S., Rantsiou K. (2019). Antilisterial effect and influence on *Listeria monocytogenes* gene expression of enterocin or *Enterococcus faecalis* in sliced dry-cured ham stored at 7 °C. J. Food Prot..

[33] Schrama D., Helliwell N., Neto L., Faleiro M.L. (2013). Adaptation of *Listeria monocytogenes* in a simulated cheese medium: Effects on virulence using the *Galleria mellonella* infection model. Lett. Appl. Microbiol..

[34] Chaturongakul S., Raengpradub S., Wiedmann M., Boor K.J. (2008). Modulation of stress and virulence in *Listeria monocytogenes*. Trends Microbiol..

[35] Sue D., Boor K.J., Wiedmann M. (2003). σ^B^-dependent expression patterns of compatible solute transporter genes o*puCA* and *lmo1421* and the conjugated bile salt hydrolase gene *bsh* in *Listeria monocytogenes*. Microbiology.

[36] Mataragas M., Rovetto F., Bellio A., Alessandria V., Rantsiou K., Decastelli L., Cocolin L. (2015). Differential gene expression profiling of *Listeria monocytogenes* in cacciatore and felino salami to reveal potential stress resistance biomarkers. Food Microbiol..

[37] Olesen I., Thorsen L., Jespersen L. (2010). Relative transcription of *Listeria monocytogenes* virulence genes in liver pâtés with varying NaCl content. Int. J. Food Microbiol..

[38] Bowman J.P., Bittencourt C.R., Ross T. (2008). Differential gene expression of *Listeria monocytogenes* during high hydrostatic pressure processing. Microbiology.

[39] Wuenscher M.D., Kohler S., Bubert A., Gerike U., Goebel W. (1993). The *iap* gene of *Listeria monocytogenes* is essential for cell viability, and its gene product, p60, has bacteriolytic activity. J. Bacteriol..

